# A Novel Approach to Segment and Classify Regional Lymph Nodes on Computed Tomography Images

**DOI:** 10.1155/2012/145926

**Published:** 2012-10-31

**Authors:** Hongmin Cai, Chunyan Cui, Haiying Tian, Min Zhang, Li Li

**Affiliations:** ^1^School of Computer Science and Engineering, South China University of Technology, Guangzhou 510006, China; ^2^State Key Laboratory of Oncology in Southern China, Imaging Diagnosis and Interventional Center, Cancer Center, Sun Yat-Sen University, Guangzhou 510060, China; ^3^Department of Automation, Sun Yat-Sen University, Guangzhou 510006, China

## Abstract

Morphology of lymph nodal metastasis is critical for diagnosis and prognosis of cancer patients. However, accurate prediction of lymph node type based on morphological information is rarely available due to lack of pathological validation. To obtain correct morphological information, lymph nodes must be segmented from computed tomography (CT) image accurately. In this paper we described a novel approach to segment and predict the status of lymph nodes from CT images and confirmed the diagnostic performance by clinical pathological results. We firstly removed noise and preserved edge details using a revised nonlinear diffusion equation, and secondly we used a repulsive-force-based snake method to segment the lymph nodes. Morphological measurements for the characterization of the node status were obtained from the segmented node image. These measurements were further selected to derive a highly representative set of node status, called feature vector. Finally, classical classification scheme based on support vector machine model was employed to simulate the prediction of nodal status. Experiments on real clinical rectal cancer data showed that the prediction performance with the proposed framework is highly consistent with pathological results. Therefore, this novel algorithm is promising for status prediction of lymph nodes.

## 1. Introduction

Accurate quantitative measurement of lymph nodal is a critical prognostic risk factor in managing rectal cancer, nasopharyngeal carcinoma [1], and other types of cancer. Morphologic characterization could serve as a quantitative criterion in differentiating benign and malignant lymph nodes [2–5]. Thus, precise segmentation of lymph node is necessary for pathological studies of rectal cancer. However, computed tomography (CT) images have some intrinsic distortions, especially near the lymph nodal boundary, thus making it difficult to evaluate the status of lymph nodes. Standard noise removal techniques such as median filtering or wavelet can be used to reduce the distortion. Though easy to implement, these standard noise reduction techniques tend to oversmooth the images and remove fine details including edges and boundaries, thus increasing the difficulty in evaluating subtle yet important information about lymph nodes such as cancer metastasis. Another challenge in using CT images to evaluate lymph nodes is the poor discrimination of benign and malignant nodes. Over the years many image processing algorithms have been developed to assist clinicians to automatically evaluate the status of lymph nodes based on some predefined criteria such as the size. However, due to the overlap in sizes between benign and malignant lymph nodes, many studies have had suboptimal performance for classifying different types of lymph nodes [6, 7]. In clinical practice, both shape and internal structure of a node are important in the differentiation between benign and malignancy [2, 3]. For example, researchers have used shape and internal structure such as smoothness and well shaped versus irregular and ill shaped or four classifications such as smooth, lobulated, spiculated, and indistinct for nodal staging using magnetic resonance images [2, 3]. In this paper we present a systematic approach to first segment “suspicious” lymph nodes and then use quantitative measurements to build an automatic classifier to discriminate benign nodes from malignant ones. Our approach includes three steps, image preprocessing, segmentation, and node classification. In order to obtain structural information of each node, we develop a revised nonlinear diffusion process. As a preprocessing step, this process adaptively weighs the homogeneity of image background to achieve a fine balance between noise removal and boundary preservation. After the preprocessing, we apply a revised snake method to segment lymph nodes. The snake model uses a repulsive force to keep the evolution from deforming to incorrect objects, thus overcoming a commonly problem with the classical models of snakes that tend to evolve to incorrect boundaries when there are neighboring objects. For each segmented node, nineteen quantitative measurements are computed and seven of them are selected to remove redundancy. Classical Support Vector Machine (SVM) model [8] is borrowing to achieve the take of nodal status classification.

This paper is organized as follows. In Section 2.1, an adaptive nonlinear diffusion method for image quality enhancement is introduced. It was followed by a semiautomatic segmentation, called repulsive snake model, which is described in Section 2.2. A new model for classification and prediction of node status is given in Section 3, which includes an recursive feature selection, classification model setting, and results verification. Experimental results using both real and simulated images are also shown in this section to demonstrate the performance of the method. Discussion and foregoing research direction are presented in Section 4.

## 2. Method

### 2.1. Nonlinear Diffusion

#### 2.1.1. Nonlinear Diffusion in Image Processing

The basic idea behind diffusion methods originated from the well-known physical phenomenon of heat transfer, which equilibrates concentration differences without creating or destroying mass. This process can be modeled by partial differential equations and their solutions describe the heat transfer at any particular time and position. Let the image domain be an open rectangle *Ω* = (0, *a*_1_)×(0, *a*_2_), let Γ ≡ ∂*Ω* be its boundary, and let the observed image *I*(*x*) be represented by a bounded function *I* : *Ω* → ℝ. Then an evolving version *u*(*x*, *t*) of *I*(*x*) with a scale time parameter *t* ≥ 0 is obtained as the solution of the following diffusion equations:

(1) $$\begin{array}{c} u_{t}=\mathrm{div}\cdot \left(D\left(\nabla u\right)\nabla u\right),\\ u\left(x,0\right)=I\left(x\right),\\ \langle D\left(\nabla u\right)\nabla u,n\rangle =0, \end{array}$$

where *I* is the initial condition at *t* = 0. For example, in the classical total variation (TV) diffusion equation [9], one can choose

(2) $$\begin{array}{c} D\left(\nabla u\right)=g\left(\left|\nabla u\right|\right)=\frac{1}{\left|\nabla u\right|}, \end{array}$$

where |∇*u*| acts as a fuzzy edge detector since pixels that have large |∇*u*| values likely belong to an edge. The role of *g*(|∇*u*|) is to adaptively control the smoothing effect. However, the diffusion coefficient *g*(·) for controlling the smoothing is based on image gradient ∇*u*, which is sensitive to the noise. Thus, the obtained image generally shows undesired “staircase effect”. To alleviate this problem, various techniques were reported [10]. Regularization by adding an additional edge preservation term is shown to be an effective solution. For example, the classical “Rudin-Osher-Fatemi” algorithm is formulated as [9]

(3) $$\begin{array}{c} u_{t}=\mathrm{div}\cdot \left(\frac{\nabla u}{\left|\nabla u\right|}\right)-\lambda \left(u-I\right),\\ u\left(x,0\right)=I\left(x\right), \end{array}$$

where the parameter *λ* is a trade-off constant in balancing image smoothing and preservation.

#### 2.1.2. Edge-Enhanced Nonlinear Diffusion

Though powerful in removing noise, the nonlinear diffusion described above has the drawback to oversmoothing the images, making it difficult to detect boundaries in the succeeding steps. To overcome this drawback, we propose a revised nonlinear diffusion method in this paper. The primary purpose of this diffusion method is to preserve edge information while removing noise in the image. We add an edge preserving term to achieve a balance between edge preservation and noise removal in an adaptive manner:

(4) $$\begin{array}{c} u_{t}=\mu \mathrm{div}\cdot \left(g\left(\left|\nabla G_{\sigma_{0}}*u\right|\right)\nabla u\right)-\frac{\left|\nabla u\right|}{\max\left|\nabla u\right|}\left(u-I\right),\\ u\left(x,y,t=0\right)=I, \end{array}$$

where *G* is a 2*D* Gaussian kernel such that

(5) $$\begin{array}{c} G_{\sigma_{0}}\left(x,y\right)=C\sigma_{0}^{-1}e^{-\left(x^{2}+y^{2}\right)/2\sigma_{0}^{2}} \end{array}$$

and *μ* of (4) represents the trade-off between smoothing and edge preservation. Our new formulation is different from the classical Rudin-Osher-Fatemi algorithm in (3) in that we use gradient magnitude |∇*u*| rather than a predefined constant to control the smoothing processing adaptively. This revised diffusion process achieves two objectives: (1) when it is near an edge, the second term will dominate the processing and thus adaptively preserve the edges from smoothing, whereas the diffusion coefficient *g*(·) is also in effect to reduce smoothness and enhance edges; (2) when it is in a homogeneous region it will reduce the noise and smooth the image.

**Figure 1:**
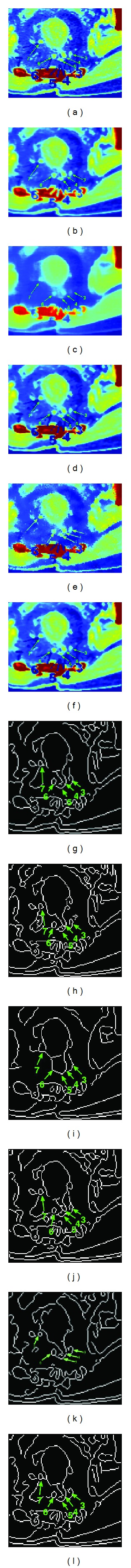
Experiments of the revised nonlinear diffusion on noise removal and edge preservation. The green arrows point to lymph nodes. (a) A real CT image, (b)–(f) smoothing results after the Perona-Malik method, TV, median filtering, bilateral filtering, and our method, respectively. The color map is used for visual comparison. (g)–(l) Edges obtained by applying the Canny operator on (a)–(f), respectively. We note that the classical methods leave “undesirable clusters” in (b)–(e), reducing the accuracy of edge detection. The proposed method produces a clean result while preserving important edge information; for example, node 6 is well preserved without any cluster around it in (f) and (l) after applying the proposed method.

To evaluate the performance of the above proposed method, we used real CT images to compare our method with some commonly used techniques such as the Perona-Malik method [11], total variation (TV) method, standard median filtering, and bilateral filtering [12].  Figure 1(a) shows an original CT image that is corrupted by noise. We then process the noisy image by the Perona-Malik method (Figure 1(b)), TV method (Figure 1(c)), median filtering (filter size = 3) (Figure 1(d)), bilateral filtering (Figure 1(e)), and our method (Figure 1(f)). To demonstrate how the proposed method preserves edges and boundaries, we next apply the Canny operator on Figures 1(a)–1(f). The corresponding results are shown in the second row of Figure 1. The images processed by the existing methods have various amounts of undesirable clutters and incorrect boundary connections, shown in Figures 1(g)–1(l). In comparison, edges obtained from our method are clean and well separated (shown in Figure 1(l)), especially around lymph nodes 3 and 6.

**Figure 2:**
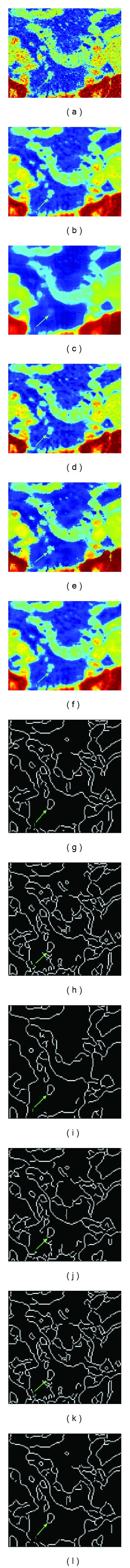
Second experiment on noise removal and edge preservation. (a) A real CT image, (b)–(f) Smoothing results after the Perona-Malik method, TV, median filtering, bilateral filter, and our method, respectively; (g)–(l) Edges obtained by applying the Canny operator on (a)–(f), respectively. The interested lymph node is labeled by green arrow. The proposed method outperformed its peers by preserving important edge information of node 1 adaptively.

We applied the same procedure on a second image, Figure 2. The interested node is labeled by green arrow and its edge information was perfectly preserved, producing a clear-cut image at Figure 2(j). In comparison, standard techniques either tend to oversmooth (Figures 2(g)–2(i), and 2(l)) or produce small clusters (Figure 2(k)). The current results markedly show that the proposed method performs better than the other three methods in terms of preserving edges while removing noise.

**Figure 3:**
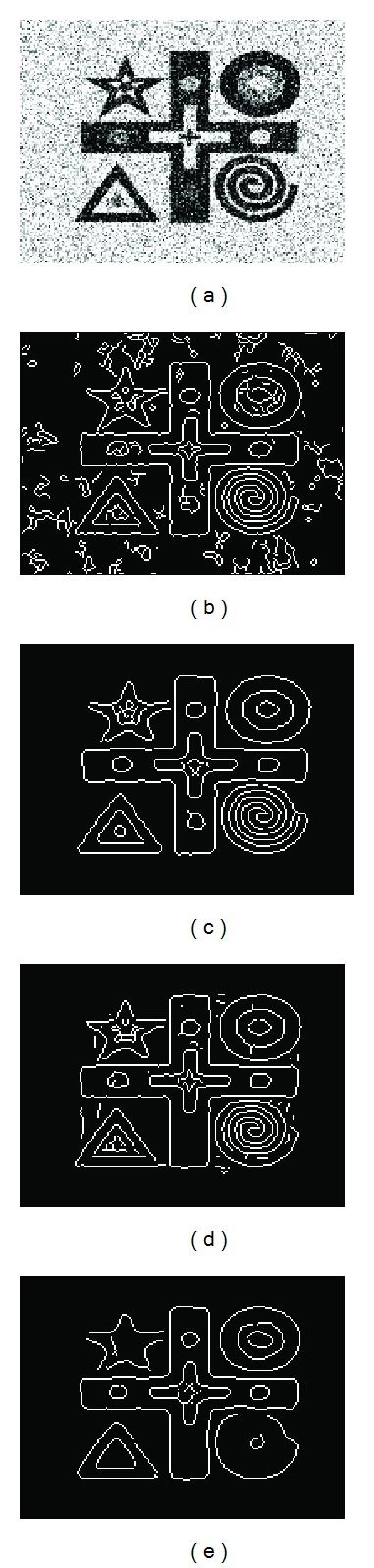
(a) A noisy image. (b) Its edge map given by Canny operator without any preprocessing. (c)–(e) are the edge maps of the given by Canny operator on the diffused images after applying the proposed method, TV, and the Perona-Malik methods, respectively.

We also apply our method to a synthetic image, (Figure 3(a)), which is degraded by additive Gaussian noise with a signal-to-noise ratio (SNR) of 9.46 dB. Because of the noise, directly applying the Canny edge detector on the image generates a suboptimal edge map, Figure 3(b). After preprocessing the noisy image by our proposed diffusion, TV, and Perona-Malik algorithms at first and then applying the same Canny edge detector, we can obtain better results, Figures 3(c)–3(e), respectively. By comparing the edge maps, we find that our method has the best performance in terms of preserving the edges while effectively reducing the noise, Figure 3(c). The TV method creates false edges (Figure 3(d)), whereas the Perona-Malik method removes some true edges (Figure 3(e)). The differences between our edge enhanced diffusion and the other two methods are fairly evident. After the preprocessing, we next use a modified snake model to segment lymph nodes from the images.

### 2.2. Repulsive Force-Based Snake Model

#### 2.2.1. Standard Snake Model

Snakes are deformable curves that can move and change their shapes to conform to object boundaries [13, 14]. The movement and deformation of snakes are controlled by internal and external forces. The aim of parametric snake or active contour, introduced by Kass et al. [13], is to minimize an energy function *E*(*C*) of a curve *C* = (*x*(*s*), *y*(*s*)) in a given image *I*. The snake is parameterized by *s* ∈ [0,1], with

(6) $$\begin{array}{c} E\left(C\right)=\int_{0}^{1}\left(\frac{1}{2}\left[\alpha {\left|C^{\prime }\left(s\right)\right|}^{2}+\beta {\left|C^{\prime \prime }\left(s\right)\right|}^{2}\right]\right)ds+\lambda E_{\text{ext}}, \end{array}$$

where *α*, *β*, and *λ* are positive coefficients and *C*′ and *C*′′ denote the first and second order derivative of *C* with respect to *s*, respectively. Here *E*_ext_ represents the external energy which generally depends on the *gradient magnitude* of the image.

A common limitation of the snake model is its inefficiency in sensing the external force, thus making it difficult to evolve to the correct boundary if its initialization is far from the legitimate object. To overcome this setback, Xu and Prince [14] presented a new external force for parametric snake model by diffusing gradient vectors of the original image. This external force is a new vector field **v** = (*u*, *v*), obtained from diffusion of the gradient vectors of a gray level or binary edge map derived form the original image. Variational minimization of such diffusion process results in the following two Euler equations:

(7) $$\begin{array}{c} \mu \nabla^{2}u-\left(f_{x}^{2}+f_{y}^{2}\right)\left(u-f_{x}\right)=0,\\ \mu \nabla^{2}v-\left(f_{x}^{2}+f_{y}^{2}\right)\left(v-f_{y}\right)=0, \end{array}$$

where *u* and *v* are the “interpolated vectors” and *f* is the edge force, usually set so that *f* = |∇*I*|^2^. It has been shown that the revised gradient vector flow (GVF) model has a much larger capture range than the original snake model and is considerably less sensitive to initialization [15, 16]. It has been confirmed to perform better than the standard snake in detecting concave boundaries [14]. However, it fails to correctly segment adjacent objects, especially when multiple objects are adjacent to each other and GVF model creates incorrect external force vectors [17, 18], thus limiting its wide application. In CT images, lymph nodes are often adjacent to nearby structures or organs. To solve such problem, we describe a new snake model that is well suited to segment adjacent objects.

#### 2.2.2. Repulsive Force-Based GVF Model

In our model we use a repulsive force to push the snakes towards their legitimate boundaries. This repulsive force can be obtained by reversing the gradient direction of neighboring objects beyond an initial curve as follows:

(8) v−={v,     if  v∈R;−v, otherwise.

Here **v** denotes the external force derived by setting **v** = ∇*G*_*σ*_0__(*x*, *y*) and *R* is the region specified in initialization. The features of GVF diffusion method, (7), are adopted to increase capture capabilities. This approach encourages the snake to deform robustly in the correct direction, even when the initial curve is placed close to other objects.

**Figure 4:**
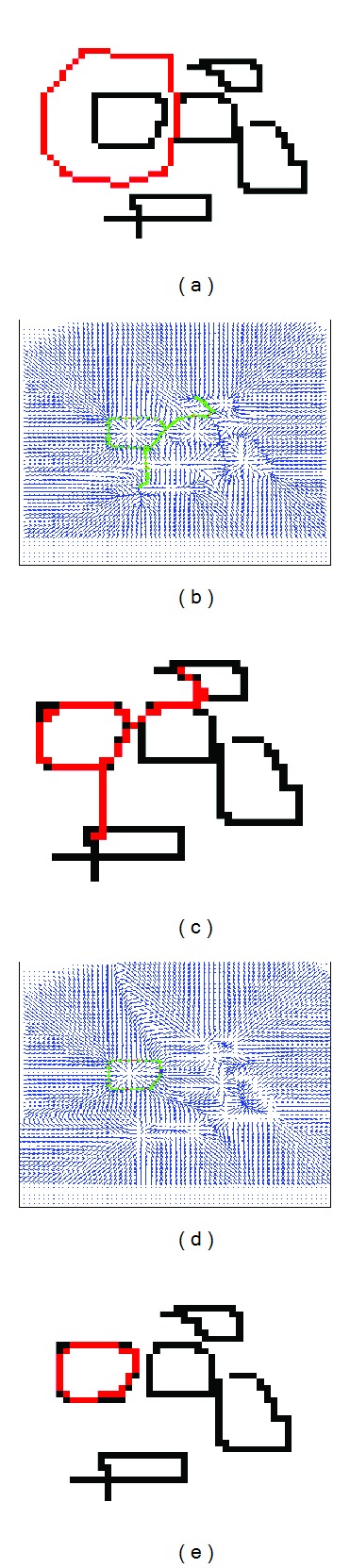
Snake deformation with repulsive force. (a) A synthetic image of multiple objects in black and initialization in red color. (b) Its associated gradient flow. (c) An initial curve at a distance from the legitimate object will deform to a wrong object. (d) However, adding external repulsive force in image (a) will revise the gradient flow and guarantee the initial curve deform to the correct object (e).

A simulation example is used to show the deformation of the snake guided by GVF and our repulsive snake model.  Figure 4(a) shows a synthetic image (in black), in which the initial curve is in red. The corresponding gradient vector field is shown in Figure 4(b).  Figure 4(c) shows the segmentation results after the GVF snake model. Due the close adjacency, the snake deforms to the nearby objects even if the initialization has correctly encircled the object of interest. The gradient flow of our repulsive snake model is shown in Figure 4(d). The final segmentation results correctly deform to the legitimate object (Figure 4(e)). The repulsive force ensures that the deformation curve evolves to the legitimate edge.

**Figure 5:**
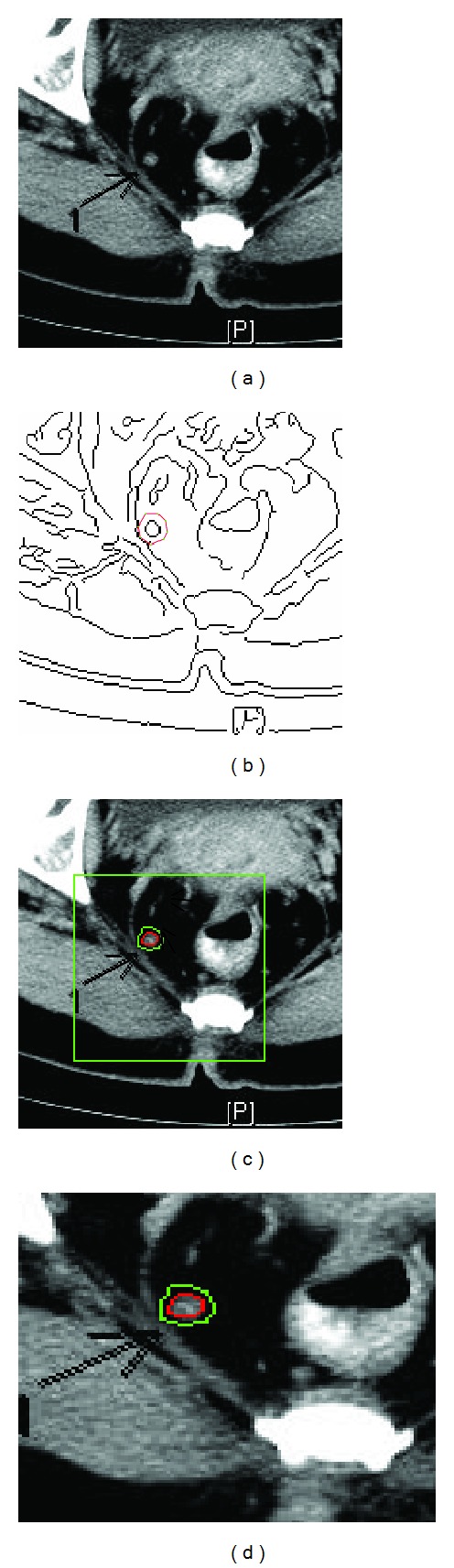
Single lymph node segmentation. (a) Original image. (b) Edge map, we use red curve to denote the initial curve for snake evolution. (c) Final segmentation result. For clarity, the interested area is zoomed out in (d).

We apply the method to an real image dataset to test its performance.  Figure 5 shows the results of using our method on a typical image of this dataset. In this example, the lymph node of interest is labeled with an arrow sign, Figure 5(a). The nonlinear diffusion preprocessing is applied to remove noise and the edges are shown in Figure 5(b). The initial curve in red line is drawn here for illustration.  Figure 5(c) shows the segmentation result after applying the new snake model. We use green color to highlight the final segmentation result. Due to the advantage of the diffusion scheme, the edge map is rather clear. It is then followed by repulsive force to obtain legitimate edge. We note that the edge structure of lymph node is important in pathology to discriminate benign lymph nodes from malignant ones. Qualitative measurements, such as fractal dimension, of accurate edge information are the key for automatic characterization of node type.

**Figure 6:**
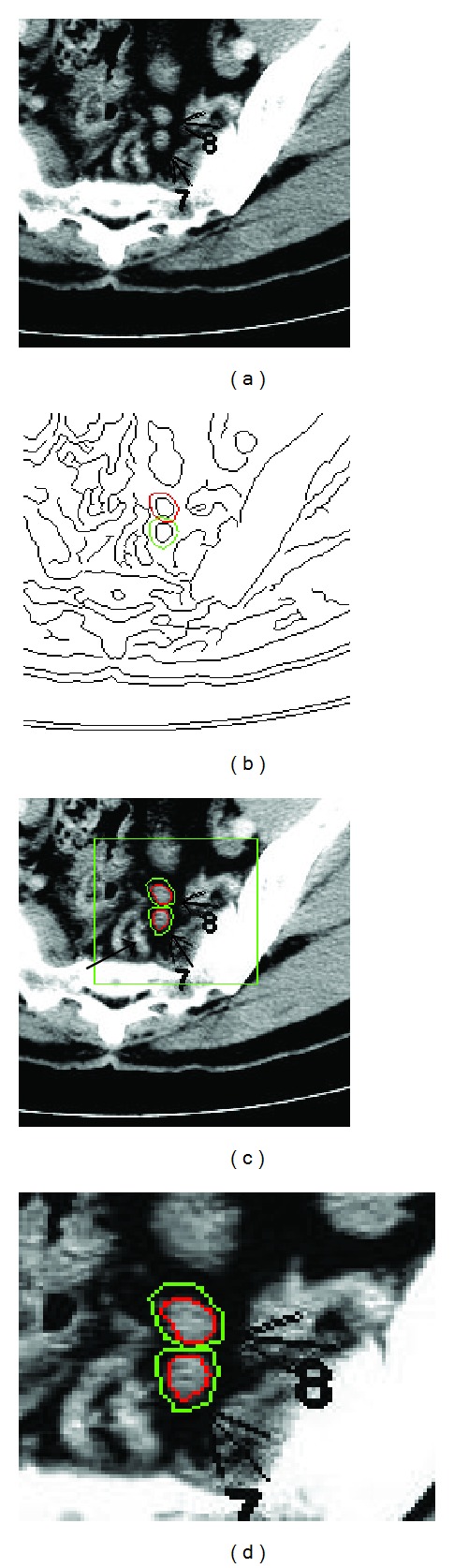
Multiple lymph nodes and their segmentation result. (a) An original CT image. (b) Edge map given by Canny method; two initial curves for snake deformation are labeled in red and green color; (c) final segmentation result. The initial curves will deform to legitimate nodes even though the initial curve is placed nearing to nearby organs. (d) The nodal area of interested is zoomed in for visual inspection.

A second example is shown in Figure 6(a) in which two lymph nodes are labeled by a radiologist using arrows 7 and 8. The edge map after the diffusion model is shown in Figure 6(b), in which the initiated curves are in different colors (red and green). The final segmentation results are displayed in Figure 6(c) and the interested area is zoomed in (Figure 6(d)) for visual inspection. We should note that although the initial curves are overlapped, the evolution can still deform to the correct lymph nodes. After segmenting the lymph nodes, we measure their quantitative features and construct a classifier about the type of each node, discussed in details below.

## 3. Lymph Nodal Classification Model

Classification of malignant and benign lymph nodes is set up using a binary pattern classification model. We use *x* ∈ **R** ^*n*^ to denote the morphological measurement of a segmented node. In this study, nineteen features of each node are computed, aiming to characterize the status as comprehensive as possible. We term these morphological features as feature vectors and use a scalar *d* ∈ {−1,1} to denote their classification label where 1 refers to the malignant node while −1 stands for the benign ones.

For such biomedical classification, three tasks are vital: effective feature extraction, accurate classification, and validation based on ground truth. We address the first two tasks by proposing a hierarchical classification model based on highly representative features obtained by an iterative feature elimination scheme. For validation, the results given by the algorithms are compared with the pathological assessment offered by expert clinicians. Each node enrolled in our study has been pathologically verified to be benign or malignant. First we search for a primary feature subset to characterize each node. Secondly, an automatic classification model based on the features is constructed to discriminate benign nodes from malignant ones.

### 3.1. Backward and Forward Feature Selection

Feature selection is one of the key components in obtaining an efficient classification model. We have included nineteen features for classification (see the appendix for details). A common problem in automatic classification is the interdependencies among features and low correlation with ground truth. To overcome such an “overfitting” problem and obtain a highly accurate and robust classifier, feature dimensionality should be reduced and an optimal features, subset, rather than all possible features, should be selected from those which are most representative for node status. We use an iterative feature elimination technique to remove the least significant features from the original feature vector and retain a minimum subset of features that can yield the best classification performance. To do so, we first obtain a training set of data, denoted by experts, and split it into two parts. We then train the classifier by using the first part to predict the second part to preserve the most prominent features. For the *j*th feature where *j* ∈ {1,19}, we randomly permute its values in the second part and then measure the accuracy of the classifier. The difference between the two values can indicate its importance. Each time the least significant feature is removed until a final necessary feature set is obtained. We call it “backward feature selection.” The obtained feature set then undergoes an opposite step by adding feature iteratively until the accuracy can no longer be increased, which is called “forward feature selection.” The backward-forward selection algorithm helps us to obtain a stable and high representative feature set. Moreover, in order to achieve a desirable sensitivity while maintaining sufficient accuracy, we measure the performance of a classifier by the following loss function:

(9) $$\begin{array}{c} \;\;\text{Loss}=||P\left(S_{1}\right)-P\left(S_{2}\right)||+2||Q\left(S_{1}\right)-Q\left(S_{2}\right)||, \end{array}$$

where *P*(·) and *Q*(·) denote the overall accuracy and sensitivity for set *S*, respectively. They are computed by

(10) $$\begin{array}{c} P\left(S\right)=\frac{\#\;\text{correctly predicted samples in set}\;S}{\#\;\text{total samples in set}\;S},\\ Q\left(S\right)=\frac{\#\;\text{True Positive in set}\;S}{\#\;\text{True Positive + \# False Negative in}\;\;S}. \end{array}$$

The detailed backward and forward feature selection steps are described in Algorithms 1 and 2, respectively. Seven quantitative parameters including fractal dimension, heterogeneity, long- and short-axis diameter, nodal density, and solidity are automatically chosen from the nineteen candidate features to compose a highly representative description of node status after the iterative feature selection method.

### 3.2. Classification Model

We use a Support Vector Machine (SVM) algorithm to classify lymph nodes [8]. SVM is based on the statistical learning theory and the Vapnik-Chervonenkis (VC) dimension. Its basic idea is to minimize the bound on generalization error rather than traditional mean square error [19–26]. SVM achieves wide applications in many area attributing to its efficiency and rigorous mathematical background. Because of its efficiency and rigorous mathematical background, SVM achieves wide applications in various fields, such as microcalcification classification in breast cancer [26–29], text classification [25, 27–29], and voice recognition [30].

In this study the prediction of lymph nodal status is considered as a binary classification problem. The features obtained from each segmented nodal image are fed into the SVM algorithm for selecting the most salient ones by minimizing the cost function defined in (9). The whole procedure is listed as follows.

In this paper *N* is the number of nodal images used for training, *M* is the number of used features during the procedure, and *y*_*i*_ is the status of the *i*th node. The above algorithm is performed between Algorithms 1 and 2 iteratively until convergence. During the training, we evaluate the performance of SVM using leave-one-out cross-validation (LOOCV) error. Then we select the feature subset with the best cross-validation performance and record the performance of the trained SVM classifier on testing samples.

### 3.3. Pathological Nodal Assessments and Nodal Matching to Obtain Ground Truth

To obtain ground truth, we use expert clinical evaluation to classify each lymph node. All visible lymph nodes on the surgical specimen have been carefully labeled and numbered by a radiologist and a surgeon. The specimen was fixed in 10% formalin for 24–48 hours. All visible lymph nodes were harvested, sliced, and stained with hematoxylin and eosin (H&E) using standard protocols. The characteristics evaluated include the nodal size and spatial correlation from the anatomical landmarks. On CT images each node has been numbered by a radiologist. The node is identified as benign or malignant by pathologists, corresponding to the serial number given on the CT images. Therefore, the same lymph node can be identified on both the CT (when visible) and surgical images, thus allowing corroboration of the findings. Nodes on surgical specimens that cannot be matched to CT findings are excluded from the study.

### 3.4. Clinical Results

**Figure 7:**
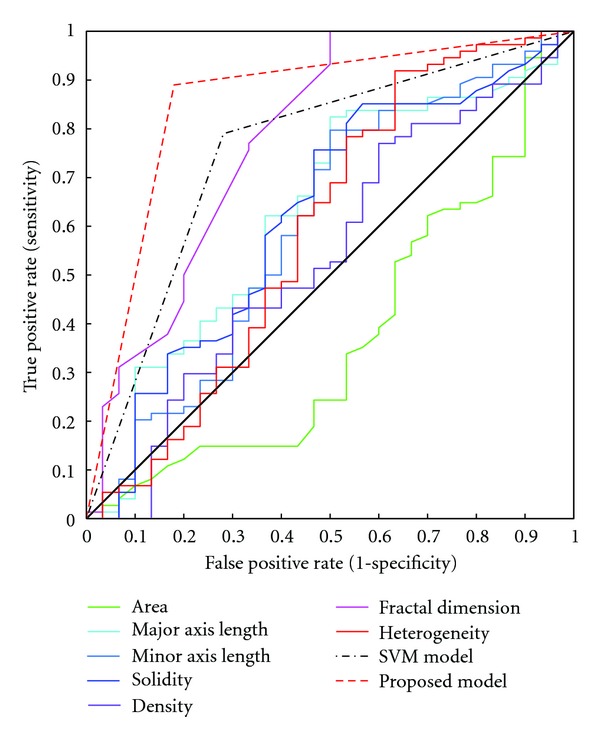
ROC curves for classification.

 To evaluate the performance of our algorithm, we tested it on real CT data of 228 patients with newly diagnosed and biopsy-proven rectal cancer between January 2007 and December 2008. Informed consents were obtained from all the eligible patients and the study was approved by the Ethical Committee of the Sun Yat-sen University. The subjects were 140 men and 88 women with an average age of 58 years old (ranging from 19 to 86 years old). These patients underwent enhanced CT examination for preoperative staging diagnosis before their total mesorectal excision. None of the patients received pre-operative chemotherapy or radiation therapy before. Using the proposed model, the prediction accuracy, sensitivity, and specificity were dramatically increased to 88%, 89%, and 82%, respectively, as compared with 78%, 79%, and 71% that yielded by the standard SVM algorithm without iterative feature selection. The receiver operating characteristic (ROC) curves for each feature (Figure 7) clearly illustrated that prediction based on the combination of seven parameters outperforms that using individual measure alone.

## 4. Discussion and Conclusions

Morphological information of lymph nodal metastasis is critical for the diagnosis and prognosis of patients with rectal cancer. Besides the size measurement which is widely used in clinical application, researchers are also interested in higher order measurements, such as heterogeneity, fractal dimension, and moments. To accurately evaluate these features, an accurate segmentation of lymph nodes is needed at first. CT images of lymph nodes are often corrupted by noises, requiring preprocess of the images before morphological analysis. Many standard techniques tend to smooth the edges while removing noise, thus inadvertently destroying structural information. This drawback significantly affects the accuracy of differentiating lymph node status based on its morphology. To address this challenge we propose to use a revised diffusion process with the aims to remove noises and simultaneously preserve boundaries of lymph nodes. Subsequently, to segment objects of interest from the background, methods such as snake models have been proposed. However, the classical GVF snake model is known to create undesired features. Therefore, we design a repulsive force-based snake method to accomplish the segmentation. The new snake method can accurately separate adjacent lymph nodes as the repulsive force guides the snake evolution to its legitimate object. With an accurate segmentation, a set of nineteen morphological features about the lymph node can be extracted. The next task is to extract pertinent information from the abundant morphological features. We develop a classifier to prune the nineteen features selected. To avoid feature overfitting, a classification model based on SVM is adopted in this work. Features that are selected automatically are fed into the proposed classifier and excellent performance results are obtained. The complete algorithms are then tested with real clinical cases and the assessment is compared with ground truth clinical diagnosis. The current model results in significantly better prediction accuracy, sensitivity, and specificity than the traditional SVM algorithm. In conclusion, the present findings strongly suggest that the morphological parameters and algorithms used herein are effective for classifying lymph node status in rectal cancer patients.

## Figures and Tables

**Algorithm 1:**
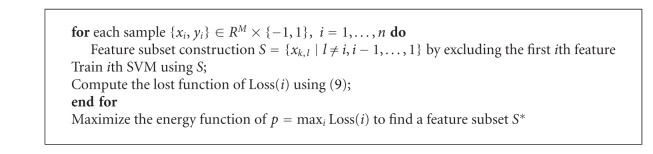
Backward selection.

**Algorithm 2:**
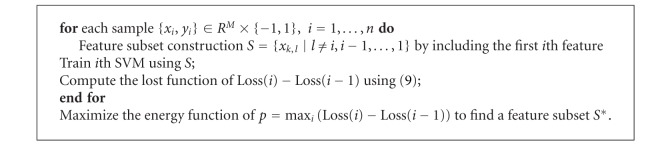
Forward selection.

## Appendix

To achieve representative characterization of a lymph node, nineteen parameters are computed automatically based on segmented image. The detailed definitions of the parameters are listed below.

1. Area: the area of the node.
2. Major and minor axes lengths: the major and minor axes of the ellipse that enclose the node with same normalized second central moments.
3. Eccentricity: the ratio of the distance between the foci of the ellipse and its major axis length. The value is used to measure the roundness of the node and is lying between 0 and 1. In degenerate cases, an ellipse whose eccentricity is 0 is actually a circle, while an ellipse whose eccentricity is 1 is a line segment.
4. Orientation: the angle between the *x*-axis and the major axis of the ellipse.
5. Convex area: the area of the smallest convex polygon that contains the node.
6. Filled area: the area of the smallest rectangle containing the node.
7. Euler number: the number of node minus the number of holes in the segmented nodal image. In most cases it equals to 1.
8. Equivalent diameter: the diameter of a circle with the same area as the node.
9. Solidity: the ratio of the area of the node and its convex area.
10. Extent: the ratio of the area of the node and its filled area.
11. Perimeter: the length of the nodal boundary.
12. Maximum and minimum gray levels: maximum and minimum gray values of the node.
13. Difference of maximum and minimum gray level: the difference in gray value of the maximum and minimum gray levels.
14. Median gray level: median gray value of a node.
15. Density: the ratio between the summation of gray value within the node and its area.
16. Heterogeneity: fraction of pixels that deviate more than a certain range (10% default) from the average intensity.
17. Fractal dimension: Minkowski dimension of the boundary of the node, computed by box-counting method.
